# Final clinical practicum shapes the transition experience and occupational commitment of newly graduated nurses in Europe—A longitudinal study

**DOI:** 10.1111/jan.15060

**Published:** 2021-10-08

**Authors:** Anu‐Marja Kaihlanen, Kia Gluschkoff, Sanna Koskinen, Leena Salminen, Camilla Strandell‐Laine, Pilar Fuster Linares, Herdís Sveinsdóttir, Natalja Fatkulina, Linda Ní Chianáin, Juliane Stubner, Helena Leino‐Kilpi, the ProCompNurse Consortium

**Affiliations:** ^1^ Finnish Institute for Health and Welfare Helsinki Finland; ^2^ Department of Nursing Science University of Turku Turku Finland; ^3^ Turku University Hospital Turku Finland; ^4^ Novia University of Applied Sciences Turku Finland; ^5^ Department of Nursing Universitat Internacional de Catalunya Sant Cugat del Vallès Spain; ^6^ Faculty of Nursing University of Iceland Reykjavik Iceland; ^7^ Faculty of Medicine Institute of Health Sciences Vilnius University Vilnius Lithuania; ^8^ School of Nursing and Midwifery National University of Ireland Galway Galway Ireland; ^9^ Institute of Health and Nursing Science Martin Luther University Halle‐Wittenberg Halle Germany

**Keywords:** clinical learning environment, final clinical practicum, graduating nursing student, newly graduated nurse, nursing, occupational commitment, transition, turnover

## Abstract

**Aims:**

The aim of the study was to examine the association between the characteristics of a nursing student’s final clinical practicum and the success of transition of newly graduated nurses (NGNs) in six European countries.

**Design:**

A longitudinal design with two data collections points (pre‐ and post‐graduate).

**Methods:**

The data were collected with an online survey between May 2018 and April 2020 from graduating nursing students (*n* = 1796) in Finland, Germany, Iceland, Ireland, Lithuania and Spain. Altogether, 642 NGNs responded to the second questionnaire 1 year after graduation. Logistic and linear regression analyses were used to examine the associations between five clinical practicum characteristics and three indicators for the success of transition (ease of transition, turnover intentions and occupational commitment). Models were adjusted for demographic and background/workplace factors and professional competence.

**Results:**

Several associations were observed between the different clinical practicum characteristics and the indicators for a successful transition. Good pedagogical atmosphere and good supervisory relationship were associated with higher likelihood of an easy transition. Good leadership style of the ward manager, good premises of nursing care on the ward and a good supervisory relationship were associated with higher occupational commitment. No consistent association with turnover intention was found.

**Conclusion:**

Having a good final clinical practicum before graduation can contribute to an easier transition experience for newly NGNs and strengthen their commitment to the nursing profession.

**Impact:**

This study adds to the limited existing knowledge about the importance of final clinical practicums in shaping the transition process and occupational commitment of NGNs. Investing in creating a good final practicum experience could help healthcare organizations engage new nursing professionals and thus alleviate the existing shortage of nurses.

## 1 INTRODUCTION

There are 27.9 million nurses in the global nursing workforce, confirming nurses as the largest occupational group in health care (WHO, 2020). An estimated 13% (3.5 million) of these nurses work in the European Union (EU) area (European Union Statistics, 2019). In several countries, the demand and supply of nurses have not met for years, and recent estimates suggest that the global shortage of nurses is 5.9 million (WHO, 2020). At the European level, the availability of a qualified health care workforce is also a major concern because it highly impacts the possibility to respond to the changing care needs of the population (European Commission Directorate‐General for Health & Food Safety, 2015). Lack of qualified nurses is a serious risk to the realization of safe and high‐quality patient care (Aiken et al., 2014; Ball et al., 2018), as has also been seen during the global COVID‐19 pandemic (Schwerdtle et al., 2020).

To tackle the nurse shortage, the number of new nursing graduates has increased in many EU countries during the past decade (European Union Statistics, 2020). However, up to every third newly graduated nurse (NGN) has high intentions to leave the nursing profession in the first years in practice (Kaihlanen, 2020; Rudman et al., 2014). Early turnover from the profession not only increases the existing nursing shortage, but also imposes significant costs (estimated at $15,000 to $40,000 per nurse) on health care organizations due to recruitment and orientation costs (e.g. Duffield et al., 2014; Hayes et al., 2012; Waldman et al., 2004). To secure the future nursing workforce, increasing understanding of how to promote NGNs’ successful transition to work life, commitment and retention in nursing profession is critical. In particular, more information is needed on the importance of nursing education and clinical practicums in this regard.

## 2 BACKGROUND

The transition of a nursing student to the role of a registered nurse is recognized as challenging and has received much attention in the literature (Duchscher, 2009; Gerrish, 2000; Kramer, 1974; Labrague & de Los Santos, 2020). The transition phase is an approximately year‐long process that proceeds in stages during which the NGN eventually achieves the confidence and comfort to work as a nursing professional (Duchscher, 2008). Across countries, the shortage of nurses has led to a situation where NGNs have to face the high expectations and demands of working life immediately after graduation, which they may feel unprepared to handle (Halpin et al., 2017; Labrague et al., 2019). NGNs are expected to be competent, manage heavy workloads and complex patient care, and quickly take responsibility and function effectively in a new role and often in a new nursing environment. This can result in stress and exhaustion (Duchscher, 2008; Halpin et al., 2017; Labrague & McEnroe‐Petitte, 2018), which, in turn, may undermine professional commitment (Hoeve et al., 2020; Raižiene & Endriulaitiene, 2007) and along with a weak professional identity (Zhang et al., 2017), contribute to NGNs’ intentions to leave the profession (Boamah & Laschinger, 2016; Butler & Johnson, 2020; Rudman et al., 2014).

Occupational commitment (also referred to as professional commitment) has repeatedly been shown to be associated with NGNs’ turnover intentions (Guerrero et al., 2017; Numminen et al., 2016) as well as actual turnover from the profession (Chang et al., 2015). Occupational commitment in nursing is conceptualized as a sense of belonging to the profession, connection to work and professional behaviours that results from the acquisition of knowledge, skills and attitudes. In addition, occupational commitment forms a moral understanding of the provision of good care, the recognition of independence, self‐regulation and responsibility in the nursing profession (García‐Moyano et al., 2019).

To date, research on the occupational commitment of NGNs has been largely focused on work‐related and organizational factors (Hoeve et al., 2018, 2020; Numminen et al., 2016), and to a less extent on the role of nursing education. However, previous research indicates that the basis for nurses’ professional values, such as commitment, is formed during nursing education (Sibandze & Scafide, 2018). Enhancing the occupational commitment of nursing students is important because having positive pre‐entry perceptions and professional motivation may enhance their later commitment and retention as NGNs (Gambino, 2010; Guerrero et al., 2017; Nesje, 2015).

Clinical practicums are an important part of nursing education and play a key role in preparing nursing students for their future work and in shaping the image of the profession and themselves as nursing professionals (Flott & Linden, 2016; Järvinen et al., 2018; Saarikoski, 2018). In particular, final clinical practicums before graduation are important for the development of nursing student’s professional identity (Marañón & Pera, 2015; Neishabouri et al., 2017), which, in turn, is associated with occupational commitment (Clements et al., 2016). Well‐implemented final clinical practicums may facilitate the professional development and practice readiness of graduating nursing students (Casey et al., 2011; Golightly et al., 2017; Kaihlanen et al., 2018; Wu et al., 2015) and have been associated with easier transition experience and lower turnover intentions among NGNs (Kaihlanen et al., 2020). Moreover, the gained practice readiness (e.g. competence) has been shown to predict NGNs’ occupational commitment (Numminen et al., 2016; Walker & Campbell, 2013).

Based on the existing knowledge, it appears that final clinical learning experiences during nursing education may reflect on NGNs’ transition experience, occupational commitment and turnover intentions. However, the evidence is scarce and studies have rarely followed students from education to work life, examining the associations in a longitudinal study design. Furthermore, although the transitional challenges and high turnover rates of NGNs are an internationally shared concern, to our knowledge, the issue has not been previously explored in a cross‐national context.

## 3 THE STUDY

### 3.1 Aims

The aim of the study was to examine the association between the characteristics of a final clinical practicum and the success of transition of NGNs in six European countries. This study is part of a larger European prospective cohort study entitled Professional Competence in Nursing (ProCompNurse).

### 3.2 Design

The study used a longitudinal design with two data collections points (pre‐ and post‐graduation) between May 2018 and April 2020. Pre‐graduation (T1) data were collected from graduating nursing students at the final stage of their studies. Because the first year in practice is typically referred to as the transition year (Duchscher, 2008), post‐graduate (T2) data were collected from NGNs 1 year after graduation.

### 3.3 Sample/participants

Participants were graduating nursing students from Finland, Germany, Iceland, Ireland, Lithuania and Spain. They were recruited using convenience sampling from educational institutions (total *n* = 45) offering nursing degree programmes leading to a nurse qualification (registered nurse or equivalent) according to the EU directives (European Commission, 2005/36/EC; European Parliament Council, 2013/55/EU). Students were eligible to participate if they were in the final semester of their studies. A total of 1796 valid questionnaires (range 64‒514 per country) were collected at T1 and a total of 642 at T2 (35‒256 per country, see Table 1). The final response rate was 41.3% (at T2 vs. T1).

**TABLE 1:** Characteristics of the participants and descriptive statistics

	*N*/%/Mean	SD
Age	25.94	6.76
Gender		
Female	571/89.8	
Male		
Country		
Finland	65/10.2	
Germany		
Iceland	35/5.4	
Ireland	68/10.6	
Lithuania	132/20.5	
Spain	74/11.5	
Work environment		
Community care/hospice/social services	146/25.4	
Hospital	231/40.2	
Emergency/intensive/perioperative care	198/34.4	
Final clinical practicum, T1 (scale 1–5)		
Nurse teacher’s pedagogical cooperation with students	3.91	0.82
Pedagogical atmosphere	4.03	0.77
Leadership style of the ward manager	3.9	0.90
Premises of nursing care on the ward	3.87	0.80
The supervisory relationships	4.14	0.81
Professional competence, T1 (scale 0–100)	61.85	14.65
Easy transition, T2		
Yes	209/52.9	
No	186/47.1	
Occupational commitment, T2 (scale 1–4)	2.50	0.48
Affective commitment	3.15	0.74
Normative commitment	2.24	0.77
Accumulated cost	2.08	0.72
Limited alternatives	2.76	0.83
Turnover intention, T2		
Yes	173/30.7	
No	391/69.3	
Same work place than final placement, T2		
Yes	94/24.4	
No	291/75.6	

Note

T1: pre‐graduate data; T2: post‐graduate data.

Abbreviation: SD, standard deviation.

### 3.4 Data collection

For T1 data collection, each educational institution named a contact person(s) to collaborate with the researchers to distribute an online survey link to eligible students to their school email. REDCap electronic data capture software hosted at the University of Turku was used (Harris et al., 2019). Alternatively, paper‐based surveys were used if the educational institution opted for this. To reach as many eligible students as possible, the educational institutions were asked whether they could allocate answering time during class and send two reminders to respondents.

For T2 data collection, an online survey was delivered to NGNs in all countries, except in Germany, with REDCap software. In Germany, the data was collected with SoSci Survey. The survey was sent to the email addresses given by the NGSs at T1 while students. Two reminders were sent to increase the response rate.

The surveys were coded at both time points with an anonymized identification code to enable comparative statistical analyses.

### 3.5 Measurements

At T1, characteristics of the students’ *final clinical practicum* were measured with the Clinical Learning Environment, Supervision and Nurse Teacher (CLES+T) evaluation scale (Saarikoski et al., 2008; Saarikoski & Leino‐Kilpi, 2002). CLES+T contain 34 items divided into four dimensions: (1) Pedagogical atmosphere (e.g. ‛The learning situations were multi‐dimensional in terms of content’); (2) Leadership style of the ward manager (e.g. ‛Feedback from the ward manager could easily be considered as a learning situation’); (3) Premises of nursing care on the ward (e.g. ‛The ward’s nursing philosophy was clearly defined’); and (4) Supervisory relationships (e.g. ‘The supervision was based on a relationship of equality and promoted my learning’). The original T‐dimension of the scale was replaced by five new items that measure the nurse teacher’s (from educational institution) pedagogical cooperation with students (Strandell‐Laine, 2019) (e.g. ‘The cooperation with the nurse teacher promoted my learning’). All the items were rated on a five‐point scale (ranging from 1 = ‘fully disagree’ to 5 = ‘fully agree’). The CLES+T has been widely used in international context to evaluate the quality of the nursing students’ clinical learning environment and shown to be valid and reliable (Henriksen et al., 2012; Saarikoski et al., 2008; Vizcaya‐Moreno et al., 2015; Warne et al., 2010). In this study, Cronbach’s alpha values for the subscales ranged from 0.81 to 0.94.

At T2, three indicators were used to measure *the success of the transition*: perceived ease of transition, turnover intention from profession, and occupational commitment.

*Ease of transition* was measured with a single item asking NGN’s perception of their transition from a student to a nurse (Kaihlanen et al., 2020). The item ‘My transition from a nursing student to a registered nurse was easy’ was rated on a five‐point scale (ranging from 1 = ‘fully disagree’ to 5 = ‘fully agree). For the analysis, the item was dichotomized into 0 (fully disagree/disagree/neither agree nor disagree) and 1 (agree/fully agree, indicating an easy transition).

*Turnover intention from profession* was measured with a single item asking: ‘How often do you plan to change from nursing into another profession outside the health sector?’. The item was answered on a four‐point scale (ranging from 1 = ‘Never’ to 4 = ‘Very often’). For the analysis, the item was dichotomized into 0 (never/quite rarely) and 1 (quite often/very often, indicating having turnover intentions).

*Occupational commitment* was measured with the Occupational Commitment Scale (OCS; Blau, 2003), which is based on the definition of organizational commitment of Meyer et al. (1993). The OCS includes 24 items under four dimensions: (1) Affective commitment (e.g. ‘I’m proud to be in the field of nursing’); (2) Normative commitment (e.g. ‘I feel an obligation to remain in nursing’); (3) Accumulated costs (e.g. ‘For me to enter another profession would require giving up a substantial investment in training’); and (4) Limited alternatives (e.g. ‘If I left nursing, I feel that I would have desirable options to pursue’). The items are rated on a four‐point scale (ranging from 1 = ‘Strongly disagree’ to 4 = ‘Strongly agree’) (Blau, 2003; Meyer et al., 1993). Multiple studies have demonstrated the validity and reliability of OCS (Blau, 2003; Blau & Holladay, 2006; Wang et al., 2012). In this study, Cronbach’s alpha values for the subscales ranged from 0.87 to 0.93.

*Demographic and background variables* included age, gender, country and work environment (community care; hospital ward; hospital unit with constant surveillance; outpatient clinic; paramedic services; hospice/palliative care; social services/non‐profit organization). Because there were very few participants working in some of the work environments, the environments were classified into three broad categories for the analysis (1 = Community care/Hospice/Social services; 2 = Hospital; and 3 = Emergency/Intensive/Perioperative care).

In addition, given the noted contribution of work environmental factors and achieved professional competence in facilitating the transition and professional commitment of NGNs (Kajander‐Unkuri et al., 2014; Missen et al., 2014; Numminen et al., 2016; Rush et al., 2019), we also asked whether *NGNs’ current work place was the same as their final clinical placement* (‘same workplace’; 1 = Yes, 0 = No) (at T2), and measured the self‐assessed professional competence of graduating nursing students (at T1). The Nurse Competence Scale (NCS) (Meretoja et al., 2004) was used, including 73 items (responded with a VAS ranging from 0 = Very low to 100 = Very high) under seven subscales. Numerous studies conducted in different countries have demonstrated the validity and reliability of NCS (Flinkman et al., 2017; Meretoja et al., 2004). In this study, Cronbach’s alpha value for the scale was 0.97.

### 3.6 Data analysis

The associations were examined using either logistic (binary outcomes) or linear (continuous outcomes) regression analysis. The analysis was performed separately for each of the five dimensions of the quality of the clinical learning environment (CLES + cooperation with the nurse teacher) and each outcome (ease of transition, turnover intentions and the four dimensions of occupational commitment). After fitting an unadjusted model (step 1), each model was next adjusted for demographic and background characteristics (age, gender, country, work environment; step 2) and then for other relevant variables (same workplace as final clinical placement and the level of professional competence; step 3). The analyses were conducted using R (version 3.6.1).

### 3.7 Validity and reliability/Rigour

The measurements selected for this study (OCS, CLES+T, NCS) have previously been used in different countries, found to be suitable for a variety of nursing environments, and have been shown to be valid and reliable (Blau, 2003; Meretoja et al., 2004; Saarikoski et al., 2008). In this study, the internal consistency of the scales was at an acceptable level (Tavakol & Dennick, 2011). As no suitable validated measure was found to measure turnover (career change) intentions outside the health sector, the item was jointly developed in the research group. The item measuring ease of transition had been reported in a previous study (Kaihlanen et al., 2020), although not validated. However, it was found to be appropriate and as no other validated measurement was found, it was selected.

## 4 RESULTS

### 4.1 Characteristics of the participants

The characteristics of the participants and descriptive statistics are presented in Table 1. Close to 90% of the participants were female. They were on average 26 years old (age ranging from 20 to 60 years) and the most common work environment was hospital. A quarter of the NGNs currently worked in their former final clinical practicum placement. One in five participants (20%) had also a previous degree in health and social care and 61% had gained work experience in health care during their studies in addition to mandatory clinical practicums. The participants rated the dimensions of the clinical practicum as consistently good, with the highest value in the dimension of supervisory relationship. About half of the participants estimated that their transition from student to nurse had been easy, and nearly one‐third had intentions to leave the profession. NGNs had a rather positive view of their overall occupational commitment, the highest value being in the dimension of affective commitment.

### 4.2 Association of the clinical practicum dimensions with the NGNs’ ease of transition, occupational commitment and turnover intentions

**FIGURE 1:**
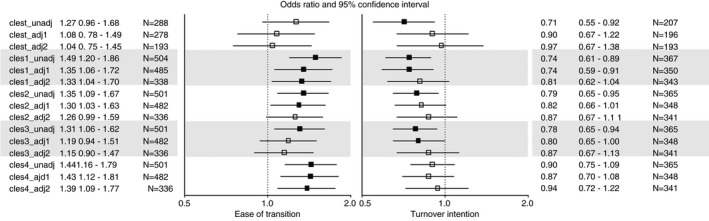
The associations of final clinical practicum with ease of transition and turnover intention by the different dimensions of clinical practicum (odds ratios and 95% confidence intervals). Statistically significant (*p* < .05) associations are shown in boldface. Clest = Nurse teacher’s pedagogical cooperation with students; cles1 = Pedagogical atmosphere; cles2 = Leadership style of the ward manager; cles3 = Premises of nursing care on the ward; cles4 = The supervisory relationships. Unadj = unadjusted model; adj1 = adjusted for age, gender, country, and work environment; adj2 = additionally adjusted for working in the same place where the clinical practicum was conducted and professional competence

**FIGURE 2:**
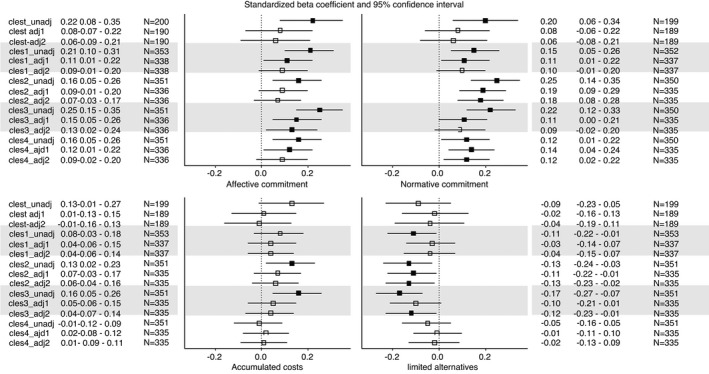
The associations of final clinical practicum with the dimensions of professional commitment by the different dimensions of clinical practicum (standardized beta coefficients and 95% confidence intervals). Statistically significant (*p* < .05) associations are shown in boldface. Clest = Nurse teacher’s pedagogical cooperation with students; cles1 = Pedagogical atmosphere; cles2 = Leadership style of the ward manager; cles3 = Premises of nursing care on the ward; cles4 = The supervisory relationships. Unadj = unadjusted model; adj1 = adjusted for age, gender, country, and work environment; adj2 = additionally adjusted for working in the same place where the clinical practicum was conducted and professional competence

The results are presented in Figure 1 (ease of transition and turnover intentions) and Figure 2 (occupational commitment). The analytic sample size ranged from *N* = 189 to *N* = 504, depending on the combination of variables that were included in the model and level of adjustment.

Good *nurse teacher’s pedagogical cooperation with students* (clest) was associated with lower likelihood of turnover intentions and higher affective and normative commitment, but associations were significant only in the unadjusted models.

A good *pedagogical atmosphere* (cles1) was associated with a higher likelihood of an easy transition and lower likelihood of turnover intentions. A good pedagogical atmosphere was also associated with higher affective and normative commitment and lower limited alternatives commitment. After adjusting for demographic variables, the associations with the ease of transition, turnover intentions and affective and normative commitment were slightly attenuated but remained significant. After additional adjustment for the ‘same workplace as practicum placement’ variable and professional competence (fully adjusted model), only the association with ease of transition remained significant.

A good *leadership style of the ward manager* (cles2) was associated higher likelihood of an easy transition, lower likelihood of turnover intentions, higher affective and normative commitment, and lower limited alternatives commitment. After adjusting for demographic variables, the associations with ease of transition and normative and limited alternatives commitment remained significant. In the fully adjusted model, the associations with normative and limited alternatives commitment remained significant.

Higher score in *premises of nursing care on the ward* (cles3) was associated with higher likelihood of an easy transition, lower likelihood of turnover intentions, higher affective, normative and accumulated costs commitment, and lower limited alternatives commitment. After adjusting for demographic variables, the associations with turnover intentions and affective, normative and limited alternatives commitment remained significant. In the fully adjusted model, the associations with affective and limited alternatives commitment were significant.

A good *supervisory relationship* (cles4) was associated with higher likelihood of an easy transition and higher affective and normative commitment. All these associations remained significant after adjusting for demographic variables, and in the fully adjusted model, the ease of transition as well as normative commitment remained significant.

In summary, the nurse teacher’s pedagogical cooperation with students was not robustly associated with any of the outcomes. The pedagogical atmosphere, the leadership style of the ward manager, and supervisory relationship were associated with ease of transition—although not necessarily after all adjustments. Although the pedagogical atmosphere and premises of nursing care on the ward were associated with turnover intention, the association was not significant if we controlled for the effects of continuing to work in the same place where the clinical practicum was conducted and professional competence. Based on a supplementary examination, it was the adjustment for the continuity in the workplace, rather than competence, which led to the attenuated association (data not shown). Finally, the leadership style of the ward manager, the premises of nursing care on the ward, and the supervisory relationship were particularly associated with the different dimensions of occupational commitment.

## 5 DISCUSSION

We examined the association between the characteristics of a graduating nursing student’s final clinical practicum and the success of transition of NGNs. We observed several associations between the different clinical practicum characteristics and the indicators for a successful transition. In the fully adjusted models, good pedagogical atmosphere and good supervisory relationship were associated with higher likelihood of an easy transition. Good leadership style of the ward manager was associated with higher normative and lower limited alternatives occupational commitment. Higher score in premises of nursing care on the ward was associated with higher affective and lower limited alternatives commitment, and a good supervisory relationship was associated with higher normative commitment.

Based on the results, none of the individual characteristics of the clinical practicum seemed to stand out clearly above the others in terms of a successful transition. Instead, the pedagogical atmosphere and premises of nursing care on the ward, the manager’s leadership practices and student’s relationship with the supervisor appeared to be equally and variably related to the ease of transition and affective, normative and limited alternatives commitment. Even if none of the practicum dimensions were consistently associated with NGNs’ turnover intentions, previous work suggests that the link between final clinical practicum and turnover intentions could be mediated by transition experience (Kaihlanen et al., 2020). Moreover, there is a well‐known link between nurses’ occupational commitment and turnover (Chang et al., 2015; Guerrero et al., 2017), but these associations between the outcome variables were not examined in this study.

In general, a good final clinical practicum was most consistently associated with the NGNs’ higher affective and normative occupational commitment, which refers to their emotional attachment to the profession and sense of loyalty and staying in the profession because of a sense of duty (Blau, 2003). The associations between the practicum and affective commitment seem particularly logical, given that the practicum is carried out under supervision, and of the components of occupational commitment, the affective component has been shown to be most strongly related to work experiences and support received from supervisors or colleagues (Allen & Meyer, 1990; Hoeve et al., 2018). However, in this study, the premises of nursing care on the ward (e.g. how clear the nursing philosophy was or whether there were problems with the flow of information related to patient care) seemed to predict NGNs’ affective commitment more than the quality of the supervision. This finding emphasizes the importance of the activities and operating culture of the entire work community for the student’s practicum.

Previous research has found a link between previously experienced job satisfaction and later affective commitment (Blau & Holladay, 2006), which supports the results of this study. Even though the final clinical practicum during nursing education cannot be considered a job, it is often the last working life experience of graduating students before their first jobs as registered nurses. Thus, a student’s good experience of the final practicum placement may well be reflected in their later emotional attachment to the profession. Affective commitment also clearly received the highest mean scores compared with the other components of occupational commitment, which has been observed in other studies as well (Numminen et al., 2016; Wang et al., 2012). This can be considered promising, since affective commitment, in particular, has previously been associated with young nurses’ intentions to leave or stay in the profession (Flinkman et al., 2008). However, previous research has also indicated that educational institutions should focus more on promoting normative commitment of students to improve NGNs’ retention (Gambino, 2010). Based on our findings, the normative commitment of NGNs could be enhanced by paying special attention to the role and practices of the ward manager and to a good supervisory relationship as supporters of student learning in their final clinical practicums.

Unlike other characteristics of clinical practicum, the nurse teacher’s pedagogical cooperation with students was not robustly associated with any of the study outcomes. This may indicate that the actions of the nurse teacher, with whom the student typically comes into contact only occasionally, are not reflected in working life in the same way as the other factors that are present daily in the student’s practicum placement. Typically, the role of the nurse teacher in the practicum is to support students in setting learning goals, bridge the gap between theoretical knowledge and practice, and participate in practicum evaluation (Papastavrou et al., 2016; Saarikoski et al., 2009). It is possible that the role of the nurse teacher in the graduation phase differs from the earlier stages of education when students may also need closer interaction and support from the teacher in various matters. On the other hand, we know very little about what kind and how central a role the nurse teacher had in different practicum placements in the participating countries.

The associations of the clinical practicum characteristics with the ease of transition, occupational commitment and turnover intensions of NGNs were often influenced by whether they had been employed in the place that was the final practicum placement. This indicates that the success of the practicum may not contribute as much to NGN’s ease of transition, commitment, or retention if the person is employed and remains in the familiar practicum setting after graduation. The finding confirms the view that familiar personnel, familiar work practices and organizational policies can be beneficial for NGNs and speed up the building of their comfort and confidence, thus facilitating the start of working life (Kaihlanen et al., 2018). In any case, creating a positive experience in the final practicum placement is crucial to increase the student’s desire to be employed in a certain unit or clinical area after graduation (Boyd‐Turner et al., 2016; Wareing et al., 2018). The creation of the best possible final clinical practicum experience can be an important recruitment tool for organizations in a situation where there is already a significant shortage of qualified nursing staff. The final clinical practicum is an integral part of nursing education in different countries; investing in its implementation can thus have a wide‐ranging impact on the nurse workforce in Europe.

### 5.1 Limitations

We were unable to make a country‐by‐country comparison because the number of participants in different countries varied significantly and was very low in some countries. Therefore, we examined data across countries and took into account possible variability between countries in the analyses. Another limitation is the fact that we do not have precise information on the possible differences in the final clinical practicums (such as length, structure, content or the role of nurse teacher) in the participating countries. The results should be interpreted with caution because multiple tests were conducted, which may have inflated the type 1 error rate (false positive). However, we examined the pattern of associations between the different dimensions of the final clinical practicum and several outcomes and did not focus on detecting specific associations. Characteristics of the practicum were defined through the dimensions of the CLES+T scale (Saarikoski et al., 2008) modified for this study. This evaluation scale was chosen as it is widely used in different countries and is known to be reliable. It is possible that by choosing other variables to describe the practicum, the results could have been different.

The analytic sample size was relatively small in some of the analyses, which may be responsible for the non‐significant findings due to lack of statistical power. It should also be noted that the problem with the use of electronic surveys as well as longitudinal research is often high nonresponse and attrition, as was also the case in this study. Some of the respondents had answered only some of the questions, in which case the analytic sample size varied according to what variables were included in the model. An attrition analysis was conducted to test whether respondents who participated in the follow‐up differed from those who had only baseline data available. Respondents lost to attrition were younger and less educated, did not have a previous health care degree or previous work experience in health care (all *p*‐values < .05), and were more likely living in Ireland, Germany, or Spain. Finally, it should be noted that it was not possible for us to control all the possible variables that may have influenced the transition of NGNs, such as team climate or quality of orientation received in the workplace.

## 6 CONCLUSION

The results of this longitudinal study suggest that having a good final clinical practicum before graduation can contribute to an easier transition experience for NGNs and strengthen their commitment to the nursing profession. In addition to adequate job orientation, it is worth investing in a well‐planned, supervised final clinical practicums that meets both the student’s individual learning needs and future career aspirations. This cannot be the sole responsibility of the practicum placement but requires sufficient resources from the education provider, as well as close co‐operation between these bodies, as both are needed to create a good practicum experience that facilitates the transition process. Professionals working in practicum placements also need more training on the transition process, how it can be promoted and how it can have an impact on the integration of new nurses into working life. The student’s transition to the role of nurse and the beginning of a nursing career can be facilitated by investing in a good learning atmosphere and providing adequate support and diverse learning opportunities on the part of the nurse manager, supervisor, and staff throughout the unit. As a result, the opportunities to engage future professionals in nursing work improve and the shortage of nurses could thus be alleviated.

## CONFLICT OF INTEREST

No conflict of interest has been declared by the authors.

## AUTHOR CONTRIBUTIONS

**Anu‐Marja Kaihlanen:** Conceptualization, Methodology, Formal analysis, Writing – Original Draft, Writing – Review and Editing, Visualization. **Kia Gluschkoff:** Conceptualization, Methodology, Formal analysis, Writing—Review and Editing, Visualization. **Sanna Koskinen:** Investigation/data collection, Writing – Review and Editing. **Leena Salminen:** Investigation/data collection, Writing – Review and Editing. **Camilla Strandell‐Laine:** Investigation/data collection, Writing—Review and Editing. **Pilar Fuster Linares:** Investigation/data collection, Writing – Review and Editing. **Herdís Sveinsdóttir:** Investigation/data collection, Writing – Review and Editing. **Linda Ní Chianáin:** Investigation/data collection, Writing—Review and Editing. **Natalja Fatkulina:** Investigation/data collection, Writing – Review and Editing. **Juliane Stubner:** Investigation/data collection, Writing – Review and Editing. **Helena Leino‐Kilpi:** Conceptualization, Investigation/data collection, Writing – Review and Editing, Supervision, Project administration, Funding acquisition. All authors have agreed on the final version of the manuscript.

## ETHICS APPROVAL

Good research practices founded on the principles of research integrity were followed (ALL European Academies, 2017). Ethical approval for the whole research project was obtained from the Ethics Committee of the University of Turku (Statement 62/2017, 11.12.2017). At T1, the research permissions were granted according to national standards from all participating educational institutions. Permission to use the scales was obtained from the copyright holders. Voluntariness and confidentiality were emphasized in the participant recruitment process. Participants signed the consent at the T1 and gave their email address for the use of T2 data collection (Regulation EU 2016/679). Consent was requested again at T2 and those expressing consent were included in the study.

### PEER REVIEW

The peer review history for this article is available at https://publons.com/publon/10.1111/jan.15060.

## Data Availability

Research data are not shared.
